# The Teaching of Anæsthetics in America

**Published:** 1910-02-12

**Authors:** 


### The Teaching of Anæsthetics in America.

From time to time, with great regularity, one or other of the American medical journals may be found to contain pessimistic views of the quantity and quality of the training which American medical students receive in the administration of anæsthetics and of the proficiency in this science which the ordinary medical man displays there. Though we too, on this side of the water, are not all satisfied with the diffusion of modern methods and teaching among the body medical, yet it would appear that we have much less cause for dissatisfaction than have the public and the profession in America. The latest reformer is Dr. Hunter Robb, the well-known gynæcologist, whose proposals to create a more efficient body of anæsthetists in the profession at large involve one suggestion at least which appears to be novel. He desires: (1) That a skilled anæsthetist, holding an appointment in the medical school as one of the faculty and in the hospital as one of the staff, be appointed at a proper salary to teach and demonstrate the administration of anæsthetics, and to administer to especially difficult and dangerous cases from the wards. (2) That in connection with a carefully prepared course of lectures on anæsthetics and their physiological action, each student be required to administer anæsthetics to dogs or other animals a certain number of times. (3) That the lecturer next take the students round the operating theatres, pointing out to them the details in connection with the administration and the method of giving anæsthetics to human beings. (4) That each student be detailed to give the anæsthetic at a certain number of operations under the guidance and criticism of the instructor and one of the more advanced pupils. (5) That the senior instead of the junior internes be detailed to administer anæsthetics, and that the latter be afforded opportunities of being present at the administrations by the former so as to learn the details by the time he reaches the second position.

From this series of recommendations one or two points are fairly obvious. One of these is that the instruction of students in this branch of professional work is much more embryonic in the medical schools across the Atlantic than it is in this country. Another is that the lecturing staff of the American schools are much better paid than are those in similar positions here, many of whom in these days of keen competition get nothing at all. At least, that is the inference suggested by the first of Dr. Robb's recommendations; for to secure a quite first-class anæsthetist to undertake these duties it is obvious that a very handsome salary would be required. It is, therefore, the more odd that so little importance should hitherto have been attached to the teaching of anæsthetics. The suggestion for a course of demonstrations and practical work upon lower animals, though unobjectionable in itself, strikes us as unnecessary, expensive, and therefore quite inexpedient. On the last point, the relative duties of senior and junior internes, it need only be said that house officers in this country have far too much work to do to be able to undertake anæsthetics in conjunction together: the waste of time would be quite prohibitive of this system. Nor would such an alteration of custom be necessary if students were properly instructed: that is the crux of the whole matter both in England and elsewhere. Moreover, the capacity to take responsibility and be worthy of it cannot be learned by deputy: to give one anæsthetic alone teaches more than to give a score under the guidance or tutelage of another, when once the student stage is overpast.

